# BTC as a Novel Biomarker Contributing to EMT *via* the PI3K-AKT Pathway in OSCC

**DOI:** 10.3389/fgene.2022.875617

**Published:** 2022-07-01

**Authors:** Ting Shen, Tianru Yang, Mianfeng Yao, Ziran Zheng, Mi He, Mengying Shao, Jiang Li, Changyun Fang

**Affiliations:** ^1^ Center of Stomatology, Xiangya Hospital, Central South University, Changsha, China; ^2^ Research Center of Oral and Maxillofacial Tumor, Xiangya Hospital, Central South University, Changsha, China; ^3^ Institute of Oral Cancer and Precancerous Lesions, Central South University, Changsha, China

**Keywords:** betacellulin, oral squamous cell carcinoma, proliferation, migration, EMT—epithelial to mesenchymal transformation

## Abstract

**Purpose:** Oral squamous cell carcinoma (OSCC) is one of the most common malignant tumors of the head and neck, while metastasis is the main cause of OSCC-related death. There is an urgent need to explore novel prognostic biomarkers and identify biological targets related to metastasis in OSCC treatment.

**Methods:** Analysis of differential expression was performed using datasets in The Cancer Genome Atlas (TCGA) and Gene Expression Omnibus (GEO). Immunohistochemistry (IHC) was conducted to assess the expression of betacellulin (BTC) in OSCC. SCC4 and CAL27 cells were used for *in vitro* experiments, in which CCK-8, transwell assays, and wounding healing assays were performed to verify the biological functions of BTC. The role of BTC in EMT was analyzed by EMT score and Western blot.

**Results:** Through the analysis of the mRNA expression profile data from TCGA database in OSCC, we found that only low expression of BTC was significantly correlated with a poor prognosis in OSCC patients. The results of IHC assays and TCGA databases showed that the expression level of BTC was related to the tumor stage, histological grade, and metastasis status. *In vitro* analysis showed that overexpression of BTC significantly suppressed the proliferation and migration of OSCC cells. Furthermore, we confirmed that BTC could affect EMT through the PI3K-AKT signaling pathway.

**Conclusion:** The overexpression of BTC suppresses the proliferation, migration, and EMT of OSCC cells *via* the PI3K-AKT pathways, leading to a better prognosis in OSCC. BTC may be used as a novel molecular marker to assess the prognosis of OSCC patients.

## Introduction

Oral squamous cell carcinoma (OSCC) is one of the most common malignant tumors of the head and neck (Siegel et al., 2018). OSCC is known for its high morbidity, mortality, and poor prognosis, with a five-year survival rate of only 60% (Siegel et al., 2012). With rapid improvements in its diagnosis and comprehensive treatment, the incidence of OSCC has declined, but the overall survival rate has only increased by 5% in the past 20 years (Chinn and Myers, 2015). OSCC has high rates of metastasis and recurrence, which greatly affect the patient prognosis and survival (Busso-Lopes et al., 2021). Therefore, it is crucial to find biomarkers and therapeutic targets for diagnosis and prognosis evaluation. However, to date, there is no clear biological target that can be used as a risk factor for OSCC metastasis and recurrence.

Epithelial–mesenchymal transition (EMT) is a cellular process wherein cells lose the morphology of the epithelial cell type and acquire the characteristics of mesenchymal cells. In OSCC, EMT is related to tumor cell migration and metastasis (Thompson et al., 2005; Lee et al., 2006; Krisanaprakornkit and Iamaroon, 2012). Recent studies have examined EMT-induced pathways that play oncogenic roles in OSCC, such as the phosphatidylinositol-4,5-bisphosphate 3-kinase (PI3K)/protein kinase B (AKT) signaling pathway, Wnt pathway, Notch pathway, and transforming growth factor-*β* (TGF-*β*)/Smad pathway (Xu et al., 2015). Recently, some second-generation sequencing data helped us further determine the relationship between OSCC and Akt phosphorylation (Chen et al., 2015). Phosphatidylinositol 3‐kinase (PI3K) activates AKT *via* the phosphorylation of membrane inositol lipids (Chen et al., 2016). Various studies have shown that PI3K-AKT pathway components are upregulated in different human malignancies (Agarwal et al., 2013; Zhang et al., 2016).

Betacellulin (BTC), the ligand of the epidermal growth factor receptor (EGFR, also known as ERBB1 and HER1), is a kind of epidermal growth factor that can promote *β*-cell regeneration (Dahlhoff et al., 2014; Rush et al., 2018; Lee et al., 2020). In previous studies, the overexpression of BTC was found to be related to a variety of cancers, including pancreatic cancer and breast cancer, and was associated with reduced survival (Olsen et al., 2018; Lee et al., 2020). However, the correlation between the expression of BTC and survival in OSCC, which would indicate its clinical significance, is still unknown.

In our study, we systematically analyzed the mRNA data of OSCC patients in TCGA database and screened out the differentially expressed gene BTC, which is related to the metastasis and prognosis of OSCC patients. According to the clinical data collected from our hospital and *in vitro* functional studies, we found that BTC may play a vital role in inhibition of OSCC progression as a tumor-suppressor gene. Furthermore, we demonstrated that BTC deficiency induces the proliferation, migration, and EMT of OSCC cells *via* the PI3K-Akt pathways, leading to a poor prognosis in OSCC. BTC may be used as a novel molecular marker to assess the prognosis of OSCC patients.

## Materials and Methods

### Ethics, Human Tissues, and Cell Lines

This study complies with the Declaration of Helsinki and was approved by the Medical Ethics Committee of the Second Xiangya Hospital of Central South University. All clinical tissue samples used for immunohistochemistry (IHC) and real-time PCR were obtained from OSCC patients who underwent surgical treatment at the Stomatological Center of the Second Xiangya Hospital of Central South University from 2013 to 2015. All patients did not receive radiotherapy and chemotherapy before and after surgery. The pathological characteristics of OSCC patients are shown in Table 1. The OSCC cell lines SCC4 and CAL27 were obtained from the Center for Molecular Medicine, Xiangya Hospital, Central South University (Changsha, China).

**TABLE 1:** Association between the clinicopathological variables and BTC expression in 38 OSCC patients.

Clinicopathological variable	No.	BTC expression		
		Negative (*n*)	Positive (*n*)	*p*
Gender	—	—	—	0.226
Male	29	16	13	—
Female	9	7	2	—
Age (years)	—	—	—	0.929
<60	25	15	10	—
≥60	13	8	5	—
Smoking	—	—	—	0.046
Yes	23	15	8	—
No	15	8	7	—
Human papilloma virus infection	—	—	—	0.162
Yes	20	10	10	—
No	18	13	5	—
N classification	—	—	—	0.004**
N2-N3	16	14	2	—
N0-N1	22	9	13	—
Tumor stage	—	—	—	0.007*
III-IV	29	21	8	—
I-II	9	2	7	—
Histological grade	—	—	—	0.01*
Poor	12	11	1	—
Well-moderate	26	12	14	—
Metastasis				
Positive	24	19	5	0.002**
Negative	14	4	10	

### Cell Culture

SCC4 and CAL27 cells were cultured in Dulbecco’s modified Eagle’s medium (DMEM)/high glucose containing 10% fetal bovine serum (FBS) and 1% penicillin–streptomycin. The cells were cultivated in a humidified 5% CO_2_ incubator.

### The Cancer Genome Atlas (TCGA) Database and GSE Dataset (GSE138206)

TCGA (https://tcga-data.nci.nih.gov/tcga/) is a public database, and the use of its datasets does not require ethical approval. The GSE dataset (GSE138206) was downloaded from the Gene Expression Omnibus (GEO) (https://www.ncbi.nlm.nih.gov/geo/query/acc.cgi?acc=GSE138206). In this study, the mRNA expression profile data and clinical follow-up data for bioinformatics analysis were downloaded from TCGA database HNSCC dataset (only the data of 330 OSCC samples and 32 matched normal oral mucosa samples were retained) and GEO database (GSE138206). Among the 330 OSCC patients, 260 patients had complete clinical follow-up data, which could be used for further survival analysis and Cox regression analysis. Based on *N* grade and *M* grade, we further divided these 260 OSCC patients into the metastasis-positive group (*n* ≥ 1 or *M* = 1) and the metastasis-negative group (*N* = 0, *M* = 0).

### Bioinformatics Analysis

In this work, only genes with a false discovery rate (FDR) < 0.05 and |log2 FC| ≥1 were selected as differentially expressed genes (DEGs) (Szklarczyk et al., 2011). Gene set enrichment analysis (GSEA) was performed with Kyoto Encyclopedia of Genes and Genomes (KEGG) gene sets (c2) or oncogenic signature gene set (c6) collections of the Molecular Signature Database v7.0 (Mootha et al., 2003; Subramanian et al., 2005). To further explore the mechanisms underlying BTC-mediated EMT, protein–protein interaction (PPI) network analysis was performed using the Search Tool for the Retrieval of Interacting Genes/Proteins (STRING) (version 11.0, https://www.string-db.org/cgi/input?sessionId=bX0xOZT5Yfvc&input_page_show_search=on) (Patil and Nakamura, 2005).

### IHC

Formalin-fixed paraffin-embedded (FFPE) tissues were embedded in paraffin. We used BTC as the primary antibody (Proteintech, Wuhan, China). Methods experiment was conducted according to histological and immunohistochemical analysis in our last study (Yao et al., 2020). The score for each slide was calculated on the basis of staining intensity and the percentage of positive cells. Immunostaining intensity was divided into four grades: 1, negative; 2, weak; 3, moderate; and 4, strong. The percentage of positively stained cells was also divided into four grades: 1, <5%; 2, 5–35%; 3, 35–75%; and 4, >75%.

### RNA Extraction and Real-Time PCR Assay

For RNA extraction, cells were plated in a six-well plate in advance. When the cells were allowed to grow to 80–100% confluence, the medium was removed, and the cells were washed with PBS. Then, 1 ml TRIzol (Invitrogen, Carlsbad, CA) was added to each well for 5 min at room temperature. The complementary DNA synthesis and quantitative real-time polymerase chain reaction were performed as described previously (Yao et al., 2020). The reverse transcription was performed according to the manufacturer’s protocol for the PrimeScript RT reagent kit (Takara, Japan). Also, GAPDH was used to normalize the expression. The primers used for real-time PCR were as follows: BTC: F: 5′-CCT​GGG​TCT​AGT​GAT​CCT​TCA-3′, R: 5′-CTT​TCC​GCT​TTG​ATT​GTG​TGG-3’; GAPDH: F: 5′-GTC​TCC​TCT​GAC​TTC​AAC​AGC​G-3′, R: 5′-ACC​ACC​CTG​TTG​CTG​TAG​CCA​A-3′.

Semiquantitative real-time PCR was performed using the SYBR Premix Ex Taq II kit (Bio-Rad, California, United States) on a Bio-Rad PCR system.

### Plasmid Transfection

293T cells in good condition and the logarithmic growth phase were used for lentivirus packaging. The cell density was controlled at approximately 80% during transfection. High-quality plasmids (PMD2.g, PAX2, Flag-plvx-sbp, and Flag-plvx-sbp-btc) were used for transfection. Puromycin was added for selection, 3 and 4 days after transfection.

### Western Blotting

A 15% sodium dodecyl sulfate-polyacrylamide gel electrophoresis (SDS-PAGE) gel was prepared the night before the experiment. The cells were plated in a six-well plate in advance, and when they reached 80% confluence, they were used for protein extraction. The original medium was removed, and the cells were washed twice with PBS. Then, 1X SDS was added, and the lysate was collected with a cell scraper and transferred to a 1.5-ml EP tube. The tube was placed at 100°C for 5 min, and the protein concentration was assessed. The same amount of total protein (20 μg per well) was separated by 15% SDS-PAGE at 80 V for 30 min and then 120 V for 60 min. Then, the proteins were transferred onto polyvinylidene fluoride membranes (Merck Millipore) at 300 mA for 60 min. Skim milk was applied to seal the blots, and the following primary antibodies were applied at 4°C overnight: AKT, pAKT (Ser 473), pAKT (Thr 308) E-cadherin, N-cadherin, vimentin (1:1000, Cell Signaling Technology, Shanghai, China), and BTC (1:1000, Proteintech, Wuhan, China). After washing three times with PBST, the secondary antibody was applied for 1 h at room temperature, and after washing three times with PBST again, the bands were visualized utilizing enhanced chemiluminescence (ECL) reagents (Pierce, United States).

### Wound Healing Assay

The cells were seeded in a six-well plate at 25–30% confluence and were cultured at 37°C and 5% CO_2_. After 3 days, a confluent cell monolayer (95–100%) was ensured, and the medium was removed from the culture dishes and washed with PBS two times. A sterile P-200 pipette tip was used to scratch the cell monolayer in a straight line in the center of the dishes. Then, the dishes were cleaned with PBS, followed by adding 2ML DMEM. Defining exact positions and focal planes, image acquisition was taken throughout 0–36 h. Quantitative data analysis was performed with Open-source software (ImageJ/Fiji).

### Transwell Cell Migration/Invasion Assay

The Matrigel was prepared at 4°C. Transwell inserts (BD Bioscience, San Jose, CA) with a filter of 8 μm were prepared for transwell cell migration/invasion assay. Matrigel of 50 μL was coated or uncoated downside surface of the transwell membrane and incubated at 37°C for 30 min for gelling. A total of 5 × 104 cells were seeded into the upper chamber with or without Matrigel, and 500 μL complete medium was added to the lower chamber. After 24-h or 48-h incubation, the cells were fixed with methanol and stained with crystal violet. Then, cells on the top surface of the membrane were wiped off. Four random fields were photographed in a phase-contrast microscope using 10 × objective. Quantitative data analysis was performed with Open-source software (ImageJ/Fiji).

### CCK-8 Assay

CCK-8 Kit was applied to evaluate cell proliferation ability (KeyGEN BioTECH, Jiangsu, China). A total of 3 × 103 cells were seeded in 96-well plates with 100 μL medium for each well. After 24, 48, 72, and 96 h, the medium in each well was removed, and then 10 μg CCK-8 solution and 100 μL medium were added into the well for 1 h in a dark environment. After that, absorbance was measured at 450 nm using a PerkinElmer’s EnSpire Multilabel Plate Reader.

### Statistical Analysis

All analyses were performed with SPSS 25.0 (SPSS Inc., United States), R (Version 4.0.3), and GraphPad Prism (version 8.0, GraphPad Software, Inc., United States). Statistical significance was determined by Student’s t-test and analysis of variance comparisons. Overall survival was assessed using the Kaplan–Meier method, and the differences in survival between the groups were compared using log-rank tests. The effect of clinicopathological factors on survival was determined with univariate and multivariate Cox proportional hazards models. Data from three independent experiments are presented as the mean ± SD. Differences with a *p*-value of <0.05 were considered statistically significant. *, **, and *** indicate *p* < 0.05, *p* < 0.01, and *p* < 0.001, respectively.

## Results

### Screening Out Genes Related to the Prognosis of OSCC

**FIGURE 1:**
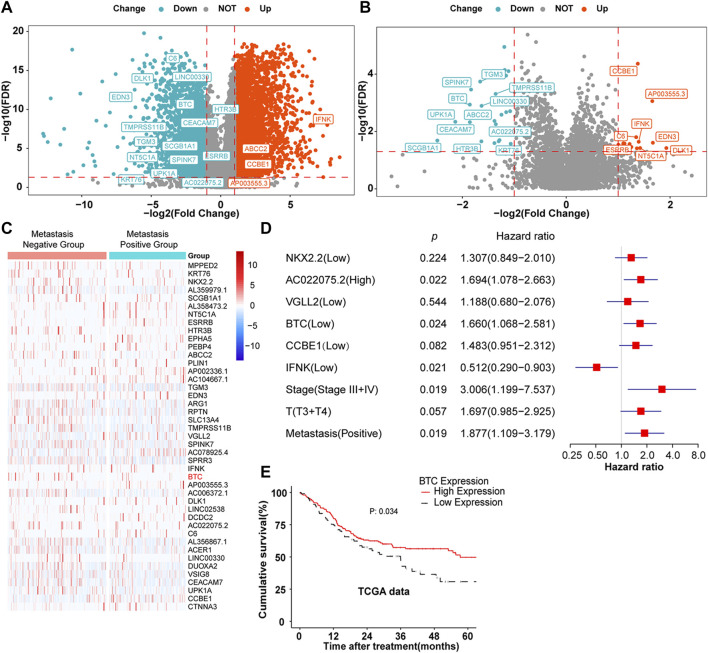
DEGs in OSCC. **(A,B)** Volcano plots were constructed using FC values and FDRs. The red points in the plot represent the overexpressed mRNAs, and the blue points indicate the downregulated mRNAs with statistical significance. **(A)** DEGs between normal and tumor tissues. **(B)** DEGs between metastasis-positive and metastasis-negative tumor tissues. **(C)** Hierarchical clustering analysis of mRNAs that were differentially expressed between metastasis-negative and metastasis-positive tissues. The normalized expression levels in the heatmaps are colored from blue to red in ascending order. **(D)** Multivariate Cox regression analysis according to gene expression. **(E)** Kaplan–Meier survival curves were performed to show the prognosis of patients with high and low expression of BTC through the analysis of the mRNA expression profile data of 260 OSCC tumor samples from TCGA database.

Through the analysis of the mRNA expression profile data in TCGA database, we found that a total of 4,626 differentially expressed genes (DEGs) were identified between 330 OSCC samples and 32 matched normal tissue specimens. Among these DEGs, 3,027 genes were upregulated in OSCC tumor tissues, whereas 1,599 genes were suppressed (Figure 1A). To further screen out the metastasis-related genes involved in OSCC, 260 patients with complete clinical follow-up data from TCGA database were divided into a metastasis-positive group (*n* = 146) and a metastasis-negative group (*n* = 113). After differential mRNA expression analysis, 43 DEGs were screened out, among which 25 genes showed low expression and 18 genes were highly expressed in the metastasis-positive group (Figures 1B,C). Furthermore, multivariate regression analysis revealed that BTC, IFNK, CCBE1, NKX2.2, VGLL2, and AC022075.2 were independent prognostic factors of OSCC among 43 DEGs (Figure 1D). Furthermore, BTC, the only tumor-suppressor gene with the largest fold change among IFNK, CCBE1, NKX2.2, VGLL2, and AC022075.2, was selected for further study, and the low expression of BTC was significantly correlated with the poor prognosis (in terms of OS) of OSCC (Figure 1E). These results indicate that BTC might be a promising biomarker for OSCC development.

### Low Expression of BTC Promotes the Development of OSCC

**FIGURE 2:**
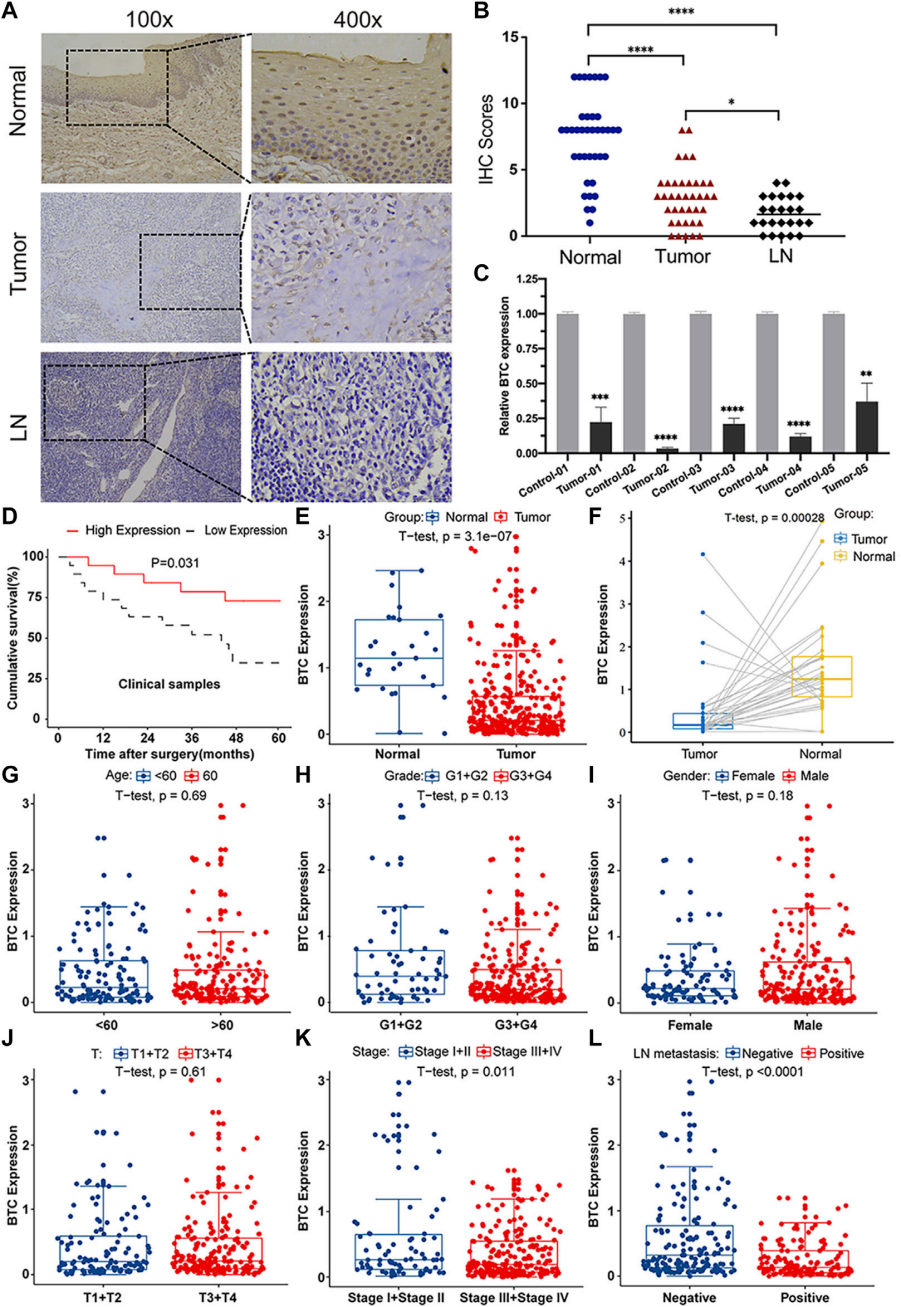
BTC expression is correlated with clinicopathological parameters in OSCC patients. **(A)** Representative images of immunohistochemical staining and **(B)** immunoreactive score of BTC in human normal mucosa samples, OSCC tissue samples, and metastatic LN samples. The experiment was repeated three times independently. Results are shown as mean ± SD. T-test, *n* = 38. **(C)** mRNA expression of BTC in normal and tumor tissues was detected by RT-PCR. **(D)** Kaplan–Meier survival curves of 38 patients from our department. **(E–L)** Analysis of 330 OSCC samples from TCGA database and 32 pairs of OSCC samples selected from TCGA database showed the comparison of **(E,F)** BTC expressed in tumor tissues and normal tissues. Correlation analysis with BTC expression and **(G)** age, **(H)** histological grade, **(I)** sex, **(J)** T category, **(K)** tumor stage, and **(L)** LN metastasis status. *p < 0.05. **p < 0.01. ***p < 0.001. ****p < 0.0001.

To further validate the role of BTC in the malignant progression of OSCC, we used IHC assays to examine the expression of BTC in 38 tissues of OSCC patients and their paired normal samples. The results showed that the expression level of BTC was remarkably lower in OSCC tissues than normal samples. In particular, the BTC expression level was significantly decreased in the lymph nodes with metastatic cancer of OSCC tissues when compared to tumor specimens (Figures 2A,B). Moreover, mRNA expression of BTC was in accordance with the results of IHC after examining 5 OSCC and paired normal samples (Figure 2C). The low expression of BTC was significantly correlated with the poor prognosis (in terms of their paired normal samples) of OSCC (Figure 2D). Furthermore, we analyzed the associations between the clinicopathological variables and BTC expression in 38 OSCC patients, and we found that the BTC expression was related to histological grade, N classification, tumor stage, and metastasis (Table 1). Through Cox proportional hazards models, we found that the low expression of BTC was an independent risk factor for prognosis in OSCC (Table 2). To further verify these results, we analyzed the expression profile data of 330 OSCC samples and 32 normal oral mucosa samples from TCGA database and found that compared to that in normal tissues, the BTC expression level in tumor tissues was low (Figures 2E,F). Furthermore, we analyzed the correlation between BTC expression and the clinical prognosis-related factors of the patients. BTC expression showed a significant correlation with patient age, tumor stage, histological grade, and lymph metastasis status (Figures 2G–L), especially in patients with lymph metastatic OSCC whose BTC levels were significantly reduced. Altogether, these results indicate that BTC may play a vital role in the malignant progression of OSCC, especially in metastasis.

**TABLE 2:** Statistical analyses of clinicopathological features associated with survival in 38 OSCC patients with the multivariate Cox proportional hazards models.

**Variable**	**Overall**	**Survival**	***p***
	**RR**	**95% CI**	
BTC expression			
Positive vs. negative	0.688	0.506–0.994	0.031*
Gender			
Male vs. female	1.124	0.903–1.868	0.329
Age (years)			
<60 vs. ≥ 60	1.341	1.002–1.849	0.021*
Smoking			
Yes vs. no	1.091	0.702–1.892	0.343
Drinking			
Yes vs. no	0.926	0.678–1.893	0.781
Tumor stage			
III-IV vs. I-II	2.449	1.567–3.459	0.038*
Histological grade			
Poor vs. well-moderate	1.311	0.912–2.466	0.061
Metastasis			
Positive vs. negative	2.612	1.232–3.635	0.043*

### BTC Expression Was Positively Related to the Proliferation, Migration, and Invasion of OSCC Cells *In Vitro*

**FIGURE 3:**
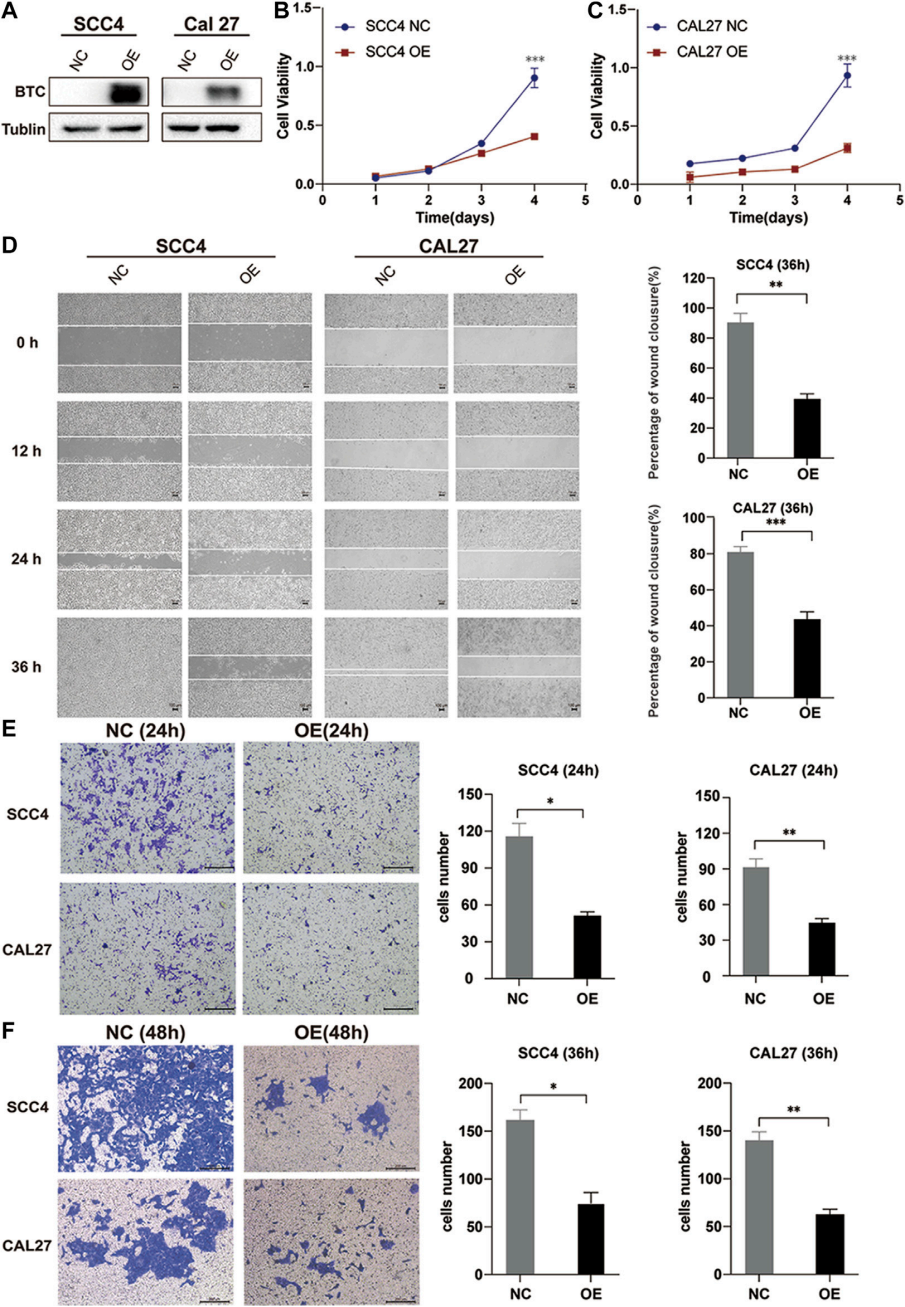
Overexpression of BTC inhibits the proliferation, migration, and invasion of OSCC cell lines. **(A)** Cancer cell transfectants of the BTC-expressing vector and empty vector control were identified in SCC4 and CAL27 cells by Western blot. **(B,C)** Overexpression of BTC inhibited cell proliferation, as indicated by the CCK-8 assay, in CAL27 and SCC4 cells. **(D)** Wound healing assay showed that overexpression of BTC inhibited CAL27 and SCC4 cell migration. **(E,F)** Transwell assays showed that the migration and invasion abilities of CAL27 and SCC4 cells were impaired after overexpression of BTC.

In order to explore the biological function of BTC in OSCC, we stably overexpressed BTC in SCC4 and CAL27 OSCC cells by lentivirus (Figure 3A). The CCK-8 assay indicated that overexpression of BTC significantly decreased cell proliferation as compared to control groups (**p < 0.01) (Figures 3B,C). Moreover, the effects of BTC on regulation of OSCC cell migration were determined by a wound healing assay. As shown in Figure 3D, the ability of cell migration was obviously inhibited after overexpression of BTC in SCC4 and CAL27 cells. In addition, the transwell assay exhibited a consistent tendency in which SCC4 and CAL27 cell migration and invasion were reduced in the overexpressed BTC group (Figures 3E,F). Collectively, these results suggest that upregulation of BTC alleviated cell growth, migration, and invasion in OSCC.

### Loss of BTC Expression Induces Epithelial and Mesenchymal Transition Changes

**FIGURE 4:**
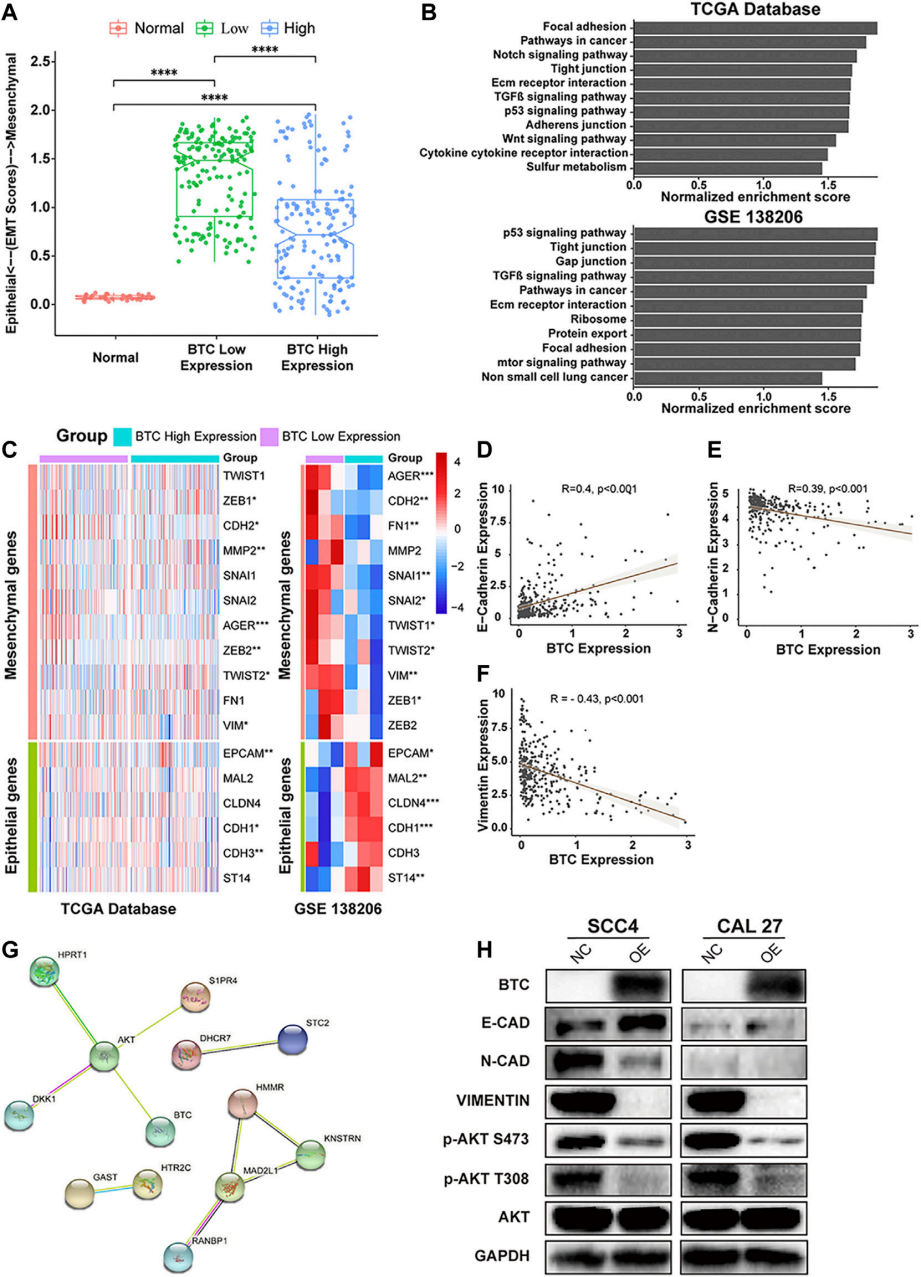
**(A)** Comparison of EMT scores in normal and tumor (in BTC low- and high-expression groups) tissue. **(B)** TCGA and GSEA showed highly regulated genes in patients with high-BTC expression versus those with low-BTC expression. **(C)** Heatmap of EMT marker expression in the BTC high- and low-expression groups. **(D–F)** Relationship between BTC and EMT markers, such as E-cadherin, N-cadherin, and vimentin. **(G)** PPI network analysis and Western blot analysis. The PPI network of the DEGs was constructed using STRING. The network nodes represent different proteins. The edges represent protein–protein associations, and the line thickness indicates the strength of the supporting data. **(H)** Protein expression level of EMT-related markers and the PI3K-AKT signaling pathway after overexpression of BTC in SCC4 and Cal27 cells.

Emerging studies have shown that EMT is an essential process that contributes to tumor invasion and metastasis in OSCC (Pastushenko and Blanpain, 2019; Chen et al., 2020). Therefore, we investigated the role of BTC in EMT of OSCC. We divided OSCC patients in TCGA database into two groups (BTC low-expression group and BTC high-expression group). As shown in Figure 4A, the BTC high-expression group had significantly decreased EMT scores when compared to BTC low-expression group. Through GSEA, the function of BTC was mainly enriched to EMT-related processes, such as tight junctions, focal adhesion, adherens junctions (Figure 4B). Moreover, whether in TCGA database or the GEO dataset (GSE 138206), we found that 17 EMT-related markers were differentially expressed between BTC low- and high-expression groups, among which 11 genes (AGER, CDH2, FH1, MMP2, SNAI1, SNAI2, TWIST 1, TWIST 2, VIM, ZEB1, and ZEB2) were related to epithelial functions and 6 genes (EPCAM, MAL2, CLDN4, CDH1, CDH3, and ST14) were associated with mesenchymal ability (Figure 4C). We also found that the expression of BTC was correlated with the expression level of multiple EMT markers, such as E-cadherin (*R* = 0.4, *p* < 0.001) (Figure 4D), N-cadherin (*R* = −0.39, *p* < 0.001) (Figure 4E), and vimentin (*R* = −0.43, *p* < 0.001) (Figure 4F).

### BTC May Inhibit the Progression of OSCC by Inhibiting the PI3K-AKT Pathway

To investigate the mechanisms underlying BTC-mediated EMT, we used STRING to construct a PPI network and found that BTC is a hub gene that can interact with AKT in the protein regulatory network of OSCC (Figure 4G). To further explore whether BTC regulated the EMT process *via* the PI3K-AKT signaling pathway in OSCC cells, Western blot analysis was performed to show that upregulation of BTC inhibited the activation of PI3K and the phosphorylation of AKT in ser473 and thr308. Furthermore, the suppression of PI3K-AKT signaling was accompanied by the altered protein expression of EMT-related markers, among which the expression of E-cadherin was significantly upregulated, whereas the expression of N-cadherin and vimentin was significantly downregulated (Figure 4H).

## Discussion

The current treatment for OSCC, especially for patients with metastasis, is still not satisfactory (Liu et al., 2021). Therefore, looking for genes related to the progression and metastasis of OSCC and exploring the mechanisms that affect the progression and metastasis of OSCC are of great significance for assessing the prognosis of OSCC patients, improving their treatment effects, and improving survival rates. In this study, we found that the lack of BTC affects EMT through the PI3K-AKT signaling pathway, leading to enhanced OSCC cell proliferation and metastasis.

In this study, we first screened out the BTC gene which was related to the progression and metastasis of OSCC through bioinformatics methods. Furthermore, we found that BTC's low expression was related to metastasis (Table 1) and poor prognosis (Figure 1E). Through Cox proportional hazards models, we found that BTC expression was an independent risk factor for OSCC (Table 2). These results suggest that BTC may play a role of tumor-suppressor gene in the progression and metastasis of oral cancer. These results were different from the previous studies, for example, Huotari et al. (1998) reported that BTC could induce a *β*-cell-like phenotype in unrelated cells by simulating *β*-cell proliferation and could also stimulate the proliferation of some cancer cells. But some scholars also found that: sequential and gamma-secretase-dependent processing of the betacellulin precursor generates a palmitoylated intracellular-domain fragment that inhibits cell growth (Stoeck et al., 2010). This difference may be related to the source of different tissues among different tumors.

EMT is a process wherein cells lose their morphology of the epithelial cell type and attain the characteristics of mesenchymal cells (Chen et al., 2015). Due to the low expression of E-cadherin and the increase in N-cadherin, adhesion reduction between OSCC cancer cells during tumor progression induces invasion and metastasis (Brabletz, 2012; Craene and Berx, 2013). In our work, we reported that overexpression of BTC could significantly decrease cell proliferation, migration, and invasion of OSCC. We also found that through GSEA, multiple genes in the BTC low-expression group were enriched in the EMT process, and the expression of BTC was obviously associated with EMT markers, such as E-cadherin, N-cadherin, and vimentin. These results illuminate that BTC may inhibit OSCC progression by regulating EMT. Furthermore, to investigate the mechanisms by which BTC regulates EMT, we found that BTC is a hub gene that interacts with AKT in the protein regulatory network of OSCC. Abundant evidence has suggested that PI3K-AKT signaling is crucial to the proliferation and survival of cancer cells with its intrinsic features of carcinogenesis. PI3K-AKT signaling is altered in approximately 30.5% of HNSCC patients (Lui et al., 2013; Lakshminarayana et al., 2018). The induction of PI3K-AKT signaling pathway components has been reported to induce the acceleration of EMT in OSCC (Wang et al., 2020; Wang and Chen, 2021). In the present study, we revealed that overexpression of BTC in OSCC could inhibit the phosphorylation of AKT by inhibiting PI3K. Meanwhile, the expression of E-cadherin was significantly upregulated, while the expression of N-cadherin and vimentin was significantly downregulated. These results indicated us that BTC may inhibit the EMT process related to OSCC progression and metastasis through the PI3K-AKT signaling pathway.

So far, the mechanism of BTC-regulated cell survivals and EMT has been poorly understood. As a ligand of EGFR, the affinity of BTC to EGFR could affect cell growth and therapy resistance in various cancers (Fan et al., 2020; Zhao et al., 2020). Furthermore, direct repression of BTC was previously reported to induce the negative regulation of EGFR-mediated survival signaling during EMT (Meyer-Schaller et al., 2018). On the contrary, we found that upregulation of BTC could inhibit cell proliferation and EMT-mediated metastasis in OSCC, which might be due to the endogenous differences between distinct tumor types. Additionally, previous studies have reported that PI3K-AKT signaling pathways could be suppressed after downregulation of EGFR in glioma, pancreatic cancer, and non-small cell lung cancer. (Milton et al., 2020; Han and Wang, 2022; Wang et al., 2022). The level of p-AKT was decreased after treatment of an EGFR antagonist (JMR-132) in ovarian cancers. Our study has found that BTC may suppress EMT in OSCC cells by downregulating the PI3K-AKT signaling. The aforementioned evidence suggests that BTC might regulate EGFR-related targets in OSCC, especially PI3K-AKT signaling. However, the role of BTC on EGFR regulation was blurred in OSCC and still needs to be further discovered.

In conclusion, low expression of BTC was associated with metastasis and poor prognosis in OSCC patients. Low expression of BTC induces the proliferation, migration, and EMT of OSCC cells *via* the PI3K-AKT pathways. BTC may be used as a novel molecular marker to assess the prognosis of OSCC patients.

## Data Availability

Publicly available datasets were analyzed in this study. These data can be found at: https://tcga-data.nci.nih.gov/tcga/.
