# Supplementary material for: BTC as a Novel Biomarker Contributing to EMT via the PI3K-AKT Pathway in OSCC
Source: Front Genet. 2022 Jul 1;13:875617. doi: 10.3389/fgene.2022.875617 (PMC9283838; doi:10.3389/fgene.2022.875617)
Supplement: Supplementary file 1 [file Presentation1.PPTX]

## Slide 1
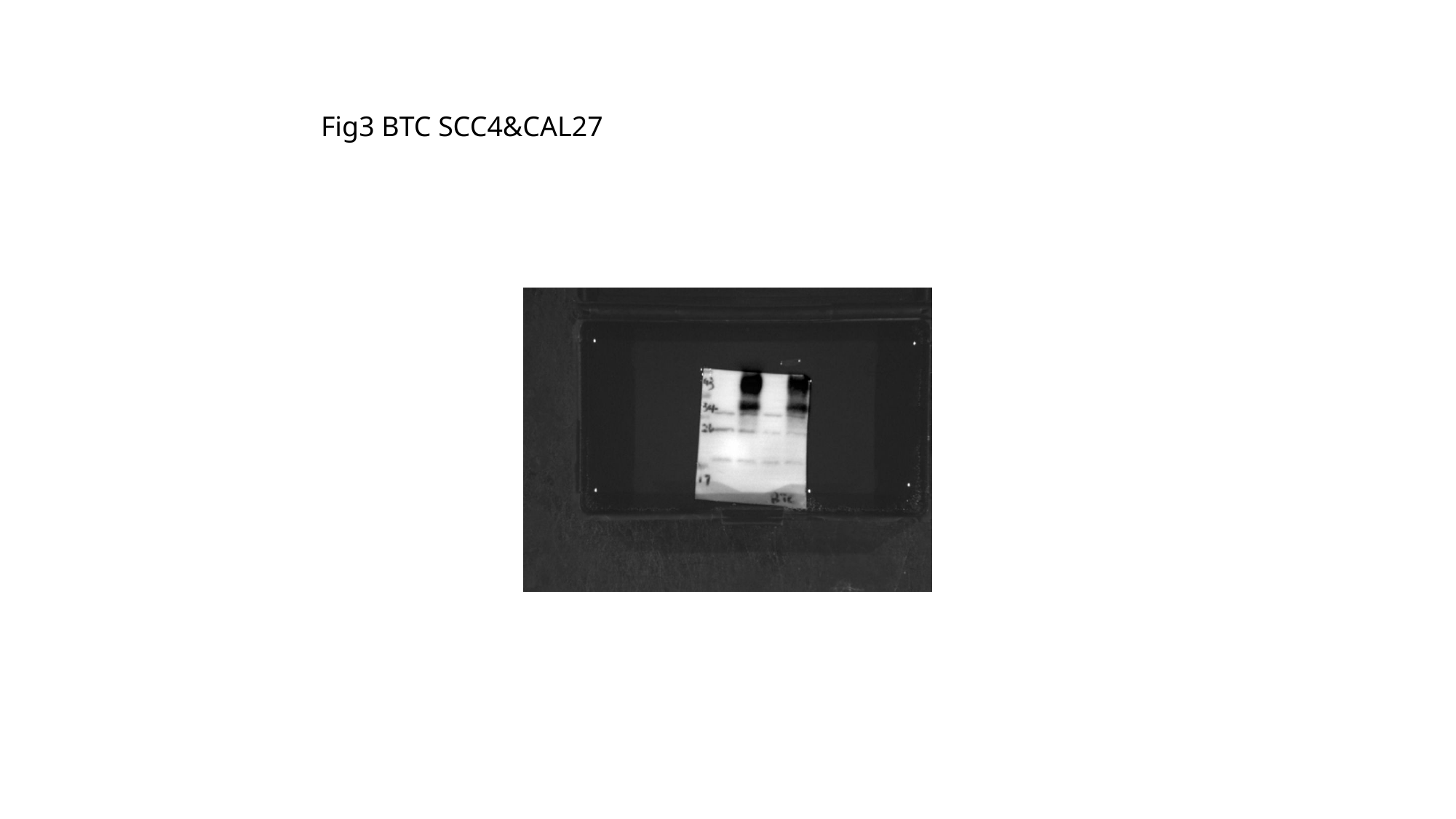

Fig3 BTC SCC4&CAL27

## Slide 2
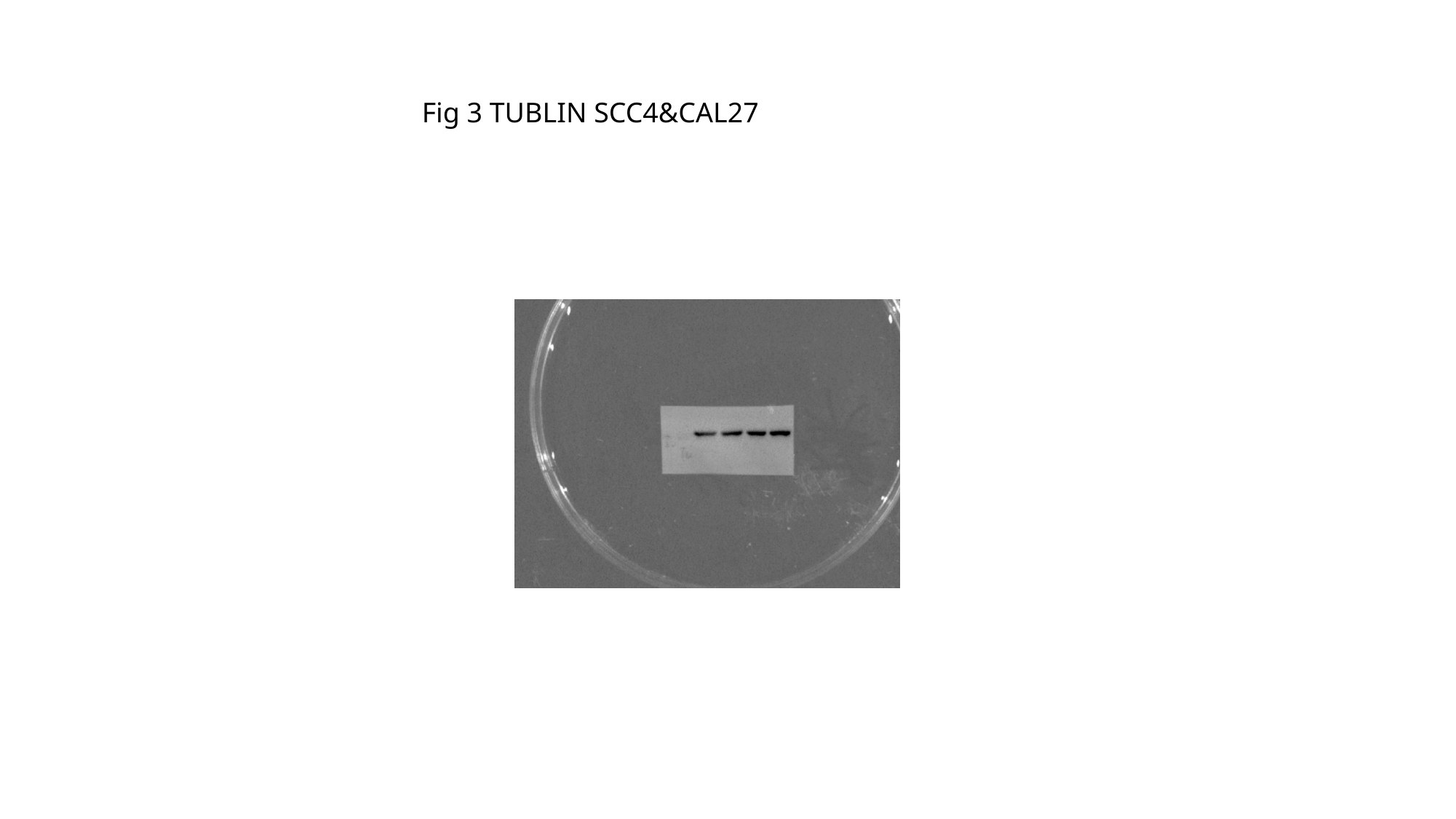

Fig 3 TUBLIN SCC4&CAL27

## Slide 3
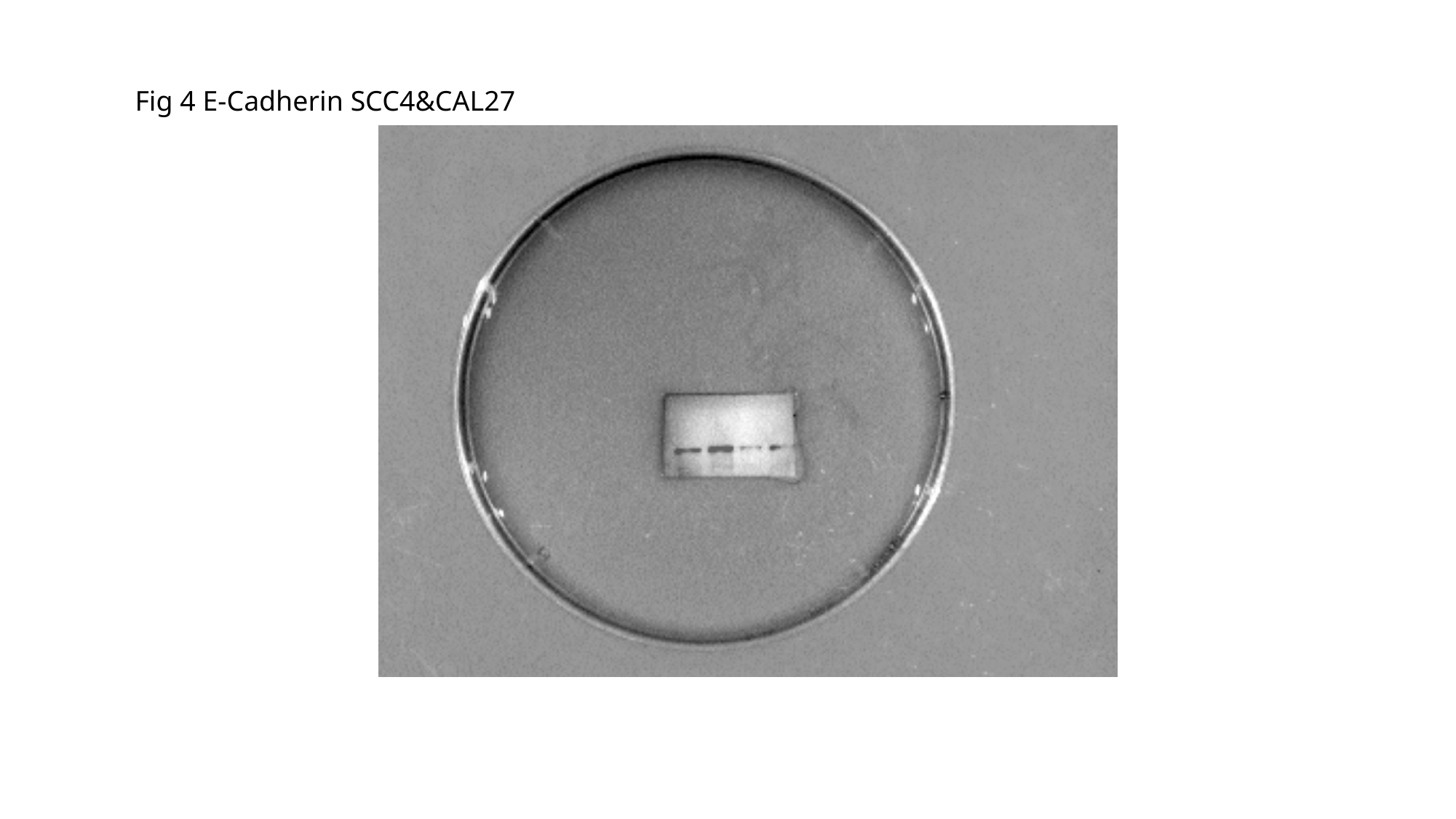

Fig 4 E-Cadherin SCC4&CAL27

## Slide 4
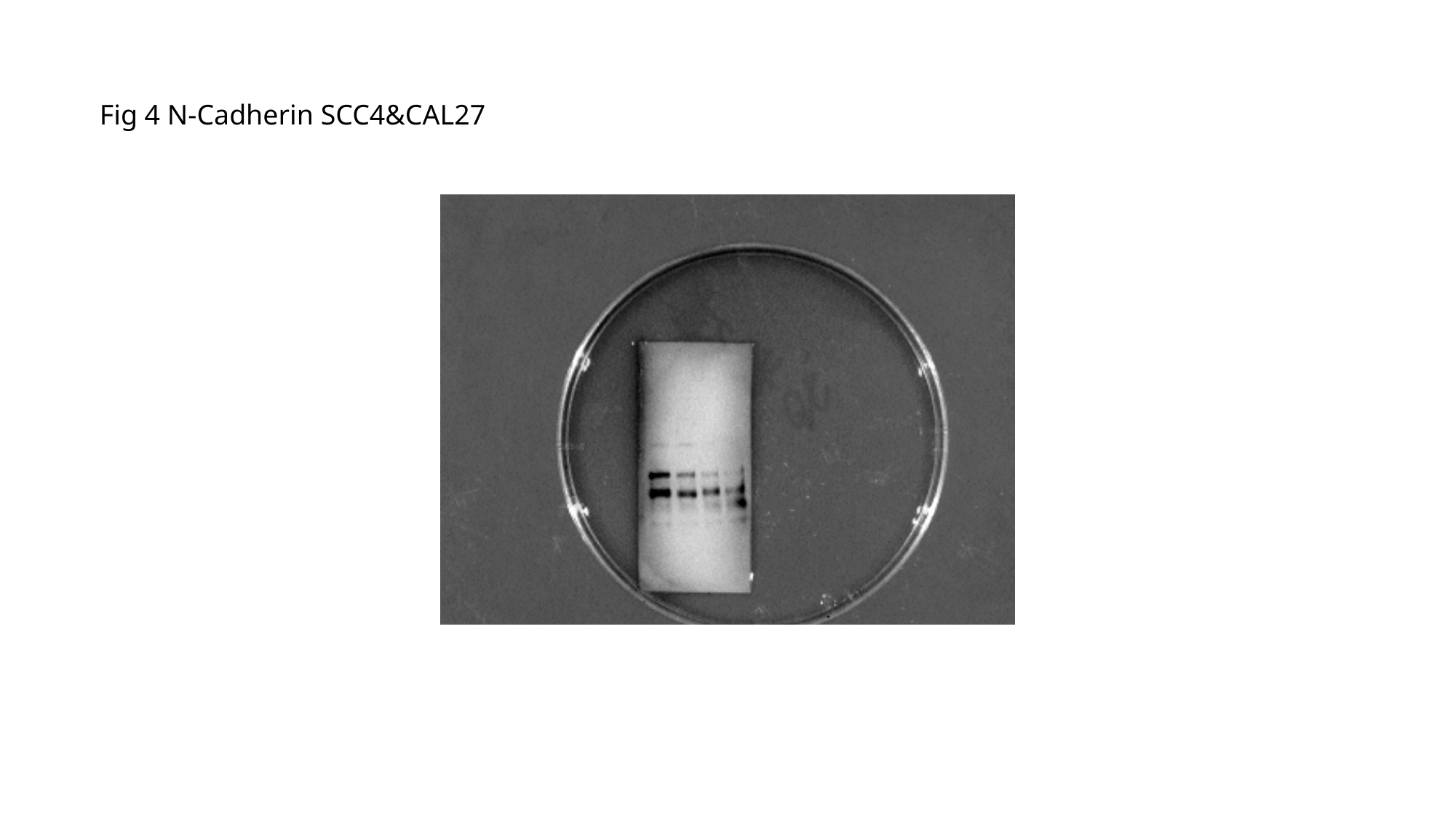

Fig 4 N-Cadherin SCC4&CAL27

## Slide 5
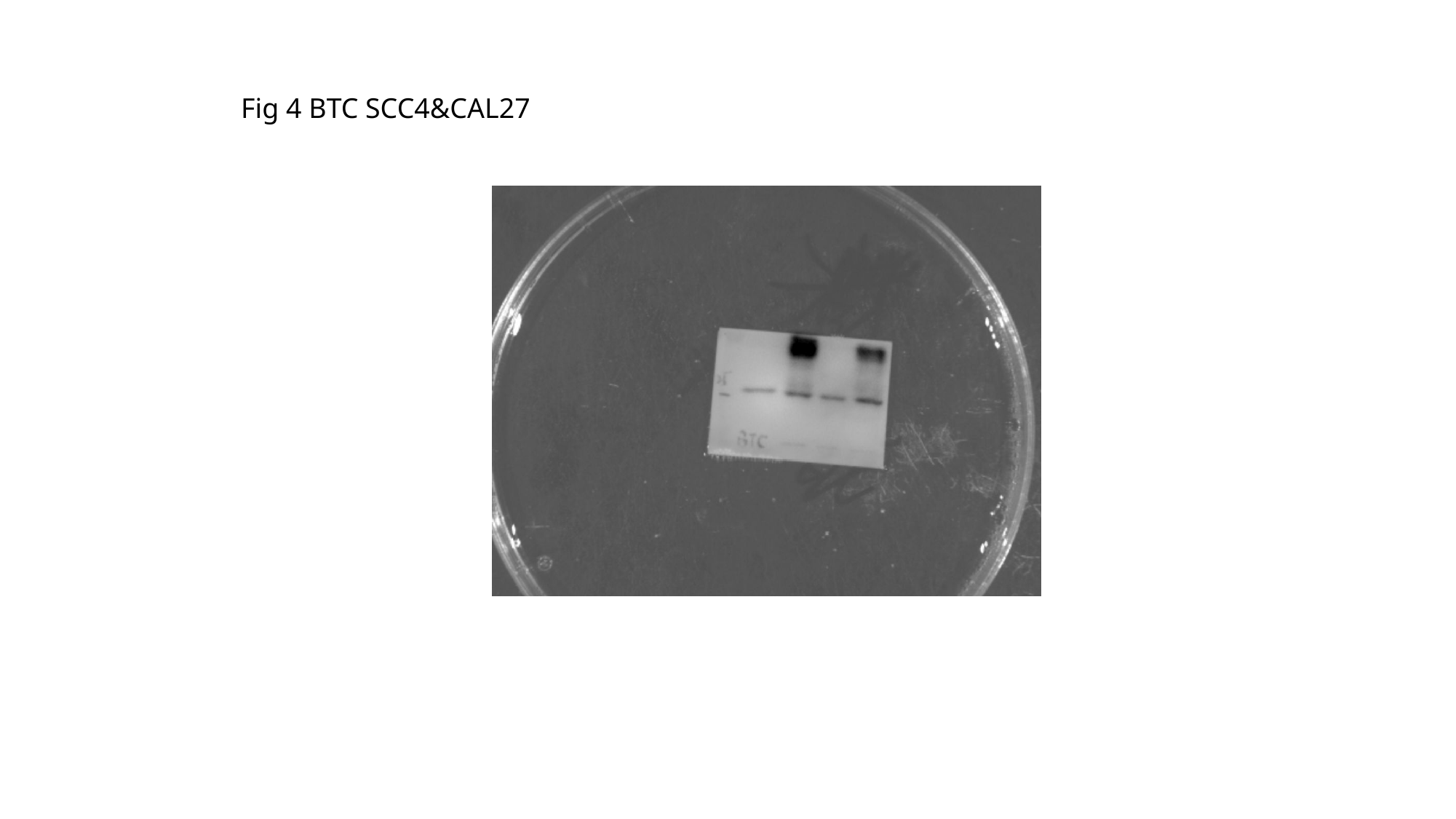

Fig 4 BTC SCC4&CAL27

## Slide 6
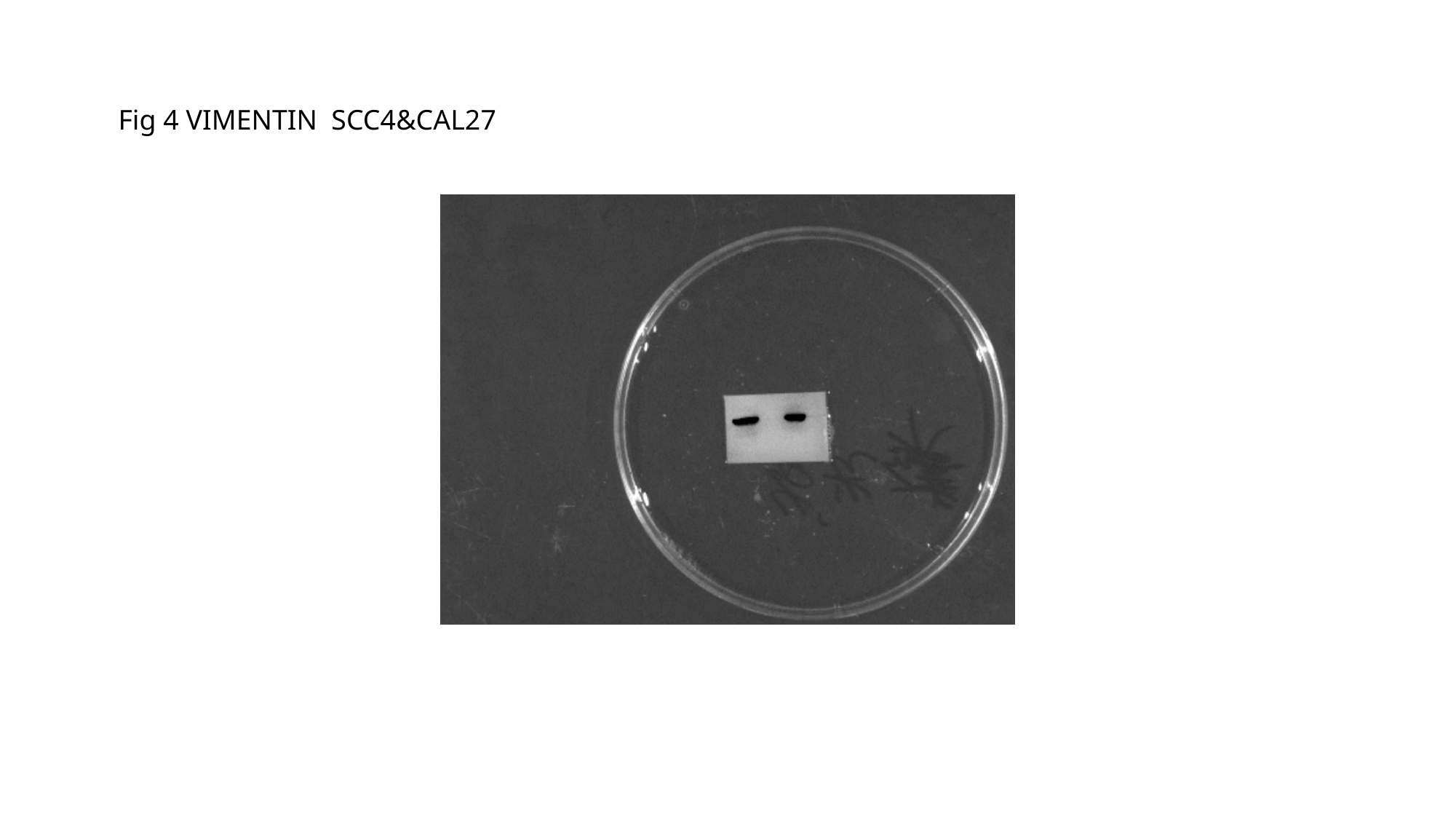

Fig 4 VIMENTIN SCC4&CAL27

## Slide 7
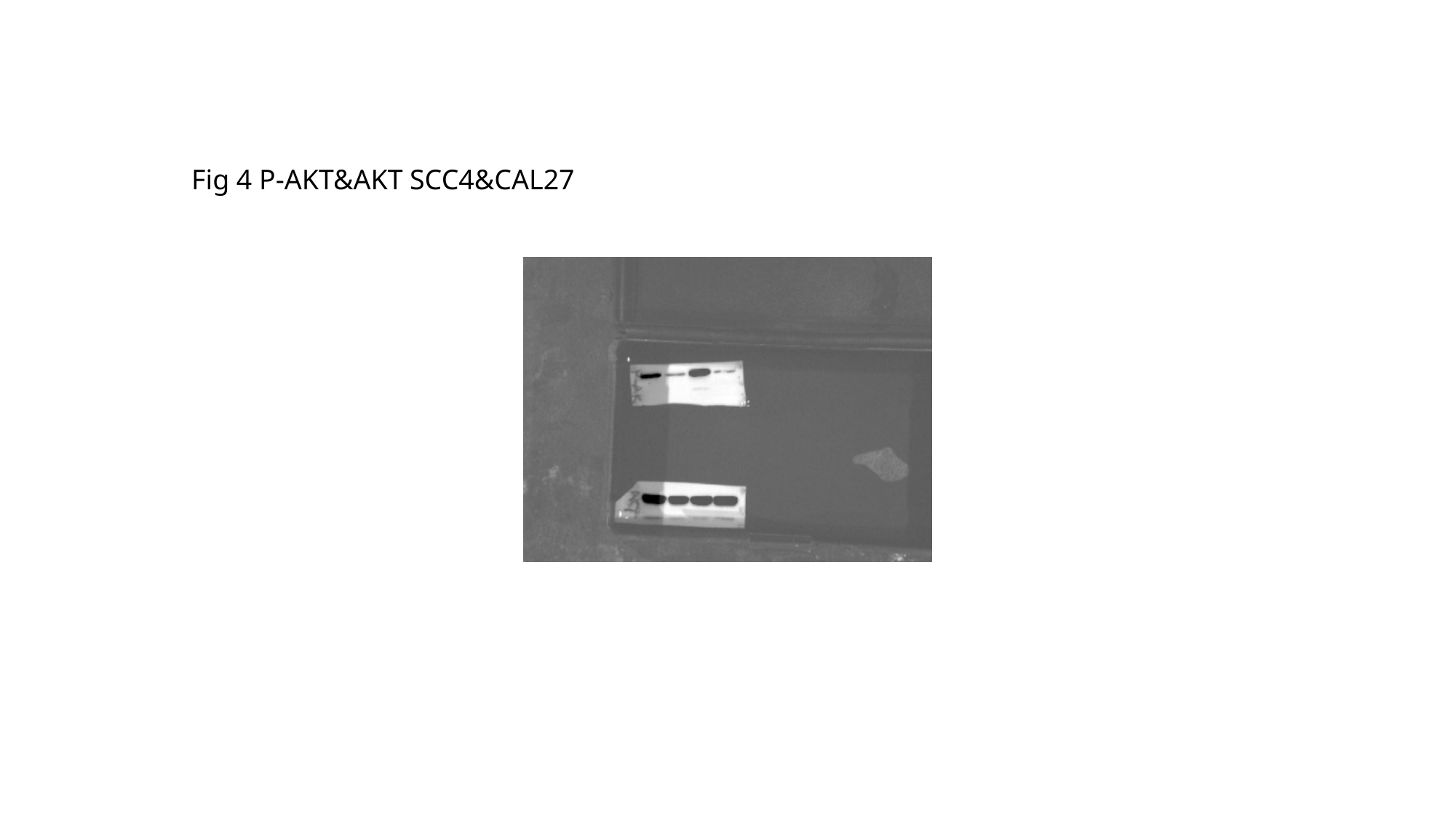

Fig 4 P-AKT&AKT SCC4&CAL27

## Slide 8
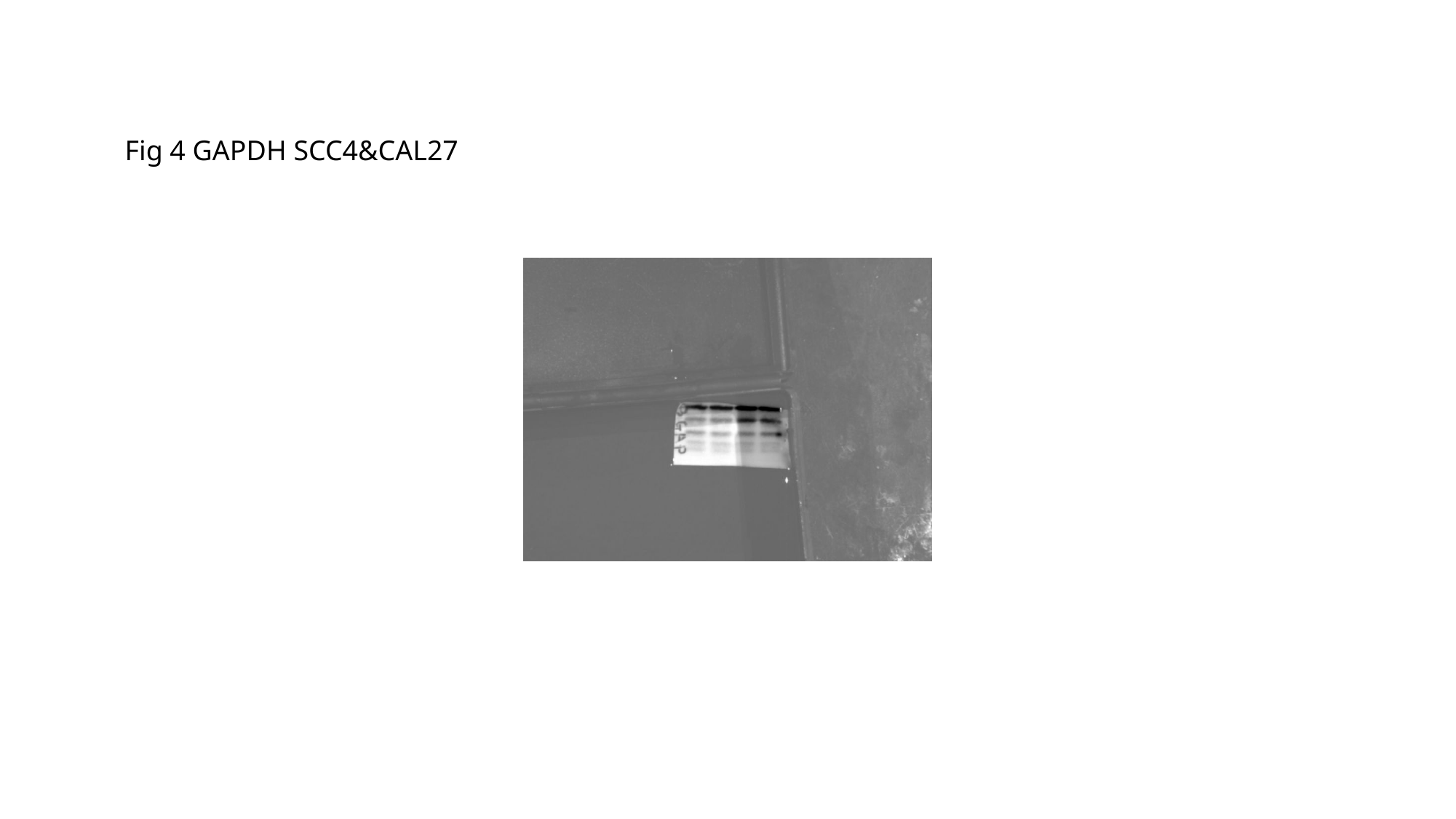

Fig 4 GAPDH SCC4&CAL27
